# Predicting neural deficits in sensorineural hearing loss from word recognition scores

**DOI:** 10.1038/s41598-022-13023-5

**Published:** 2022-06-23

**Authors:** Kelsie J. Grant, Aravindakshan Parthasarathy, Viacheslav Vasilkov, Benjamin Caswell-Midwinter, Maria E. Freitas, Victor de Gruttola, Daniel B. Polley, M. Charles Liberman, Stéphane F. Maison

**Affiliations:** 1 Eaton-Peabody Laboratories, Massachusetts Eye & Ear, 243 Charles Street, Boston, MA 02114-3096 USA; 2 Department of Otolaryngology - Head and Neck Surgery, Harvard Medical School, Boston, MA USA; 3 Department of Biostatistics, Harvard T.H. Chan School of Public Health, Boston, MA USA; 4 Present Address: Department of Communication Science and Disorders, University of Pittsburgh, Pittsburgh, PA USA

**Keywords:** Auditory system, Cochlea, Inner ear, Predictive markers

## Abstract

The current gold standard of clinical hearing assessment includes a pure-tone audiogram combined with a word recognition task. This retrospective study tests the hypothesis that deficits in word recognition that cannot be explained by loss in audibility or cognition may reflect underlying cochlear nerve degeneration (CND). We collected the audiological data of nearly 96,000 ears from patients with normal hearing, conductive hearing loss (CHL) and a variety of sensorineural etiologies including (1) age-related hearing loss (ARHL); (2) neuropathy related to vestibular schwannoma or neurofibromatosis of type 2; (3) Ménière’s disease; (4) sudden sensorineural hearing loss (SSNHL), (5) exposure to ototoxic drugs (carboplatin and/or cisplatin, vancomycin or gentamicin) or (6) noise damage including those with a 4-kHz “noise notch” or reporting occupational or recreational noise exposure. Word recognition was scored using CID W-22 monosyllabic word lists. The Articulation Index was used to predict the speech intelligibility curve using a transfer function for CID W-22. The level at which maximal intelligibility was predicted was used as presentation level (70 dB HL minimum). Word scores decreased dramatically with age and thresholds in all groups with SNHL etiologies, but relatively little in the conductive hearing loss group. Discrepancies between measured and predicted word scores were largest in patients with neuropathy, Ménière’s disease and SSNHL, intermediate in the noise-damage and ototoxic drug groups, and smallest in the ARHL group. In the CHL group, the measured and predicted word scores were very similar. Since word-score predictions assume that audiometric losses can be compensated by increasing stimulus level, their accuracy in predicting word score for CHL patients is unsurprising. The lack of a strong age effect on word scores in CHL shows that cognitive decline is not a major factor in this test. Amongst the possible contributions to word score discrepancies, CND is a prime candidate: it should worsen intelligibility without affecting thresholds and has been documented in human temporal bones with SNHL. Comparing the audiological trends observed here with the existing histopathological literature supports the notion that word score discrepancies may be a useful CND metric.

## Introduction

Recent studies on aging and noise exposure from animal models and human temporal bones show that outer hair cell loss accompanies, or is preceded by, cochlear nerve degeneration (CND), a loss of synaptic connections between the inner hair cells and a subset of auditory-nerve fibers^1–3^. This type of neural loss does not elevate behavioral or electrophysiological thresholds until it becomes extreme^4–6^, partly because the most vulnerable cochlear neurons to both noise and aging do not contribute to threshold detection in quiet^7,8^. However, the silencing of cochlear neurons degrades auditory processing and may translate into a variety of perceptual abnormalities including speech discrimination difficulties, particularly in noisy environments^3,9–12^. CND may also be key to the genesis of other perceptual anomalies associated with sensorineural hearing loss (SNHL) including hyperacusis and tinnitus, via an induction of central gain adjustment secondary to loss of afferent input to the auditory central nervous system^13–18^.

Thus, audiology best practices for hearing evaluation in adults, i.e. a standard audiogram and a word-recognition task in quiet^19^, may not fully assess the hearing impairment associated with CND. While a number of clinical centers have enhanced evaluation protocols (e.g., speech-in noise testing, evaluation of extended high frequencies), particularly in patients reporting difficulty in noisy environments despite normal or near-normal audiometric thresholds, retrospective studies of CND must rely on traditional audiometric data.

One tool of particular interest is the speech intelligibility curve (SIC), which describes the cumulative distribution of useful speech information as word presentation level is increased^20^. This sigmoidal performance-intensity function is computed from the patient’s audiogram combined with a transfer function appropriate to the speech material. In absence of multiple word presentation levels, the SIC can be a useful predictor of the maximal word recognition score (WRS). However, the assumptions behind the SIC treat a hearing loss as if it were simply a frequency-specific attenuation of the incoming speech sounds. This is largely true for a conductive hearing loss (CHL), i.e. one due to dysfunction in the sound transmission apparatus of the external and middle ears. However, for sensorineural hearing loss (SNHL), i.e. impairments arising from pathologies in the transduction of sound-induced vibration by the inner ear’s sensory cells into action potentials in cochlear nerve fibers, the pathophysiology is much more complex and cannot, in general, be corrected by simply increasing stimulus level.

In SNHL, the elevation of pure-tone thresholds is typically due to loss of, or damage to, the outer hair cells, which normally function as biological motors, amplifying the sound-evoked vibrations of the sensory epithelium^21^. In addition to threshold elevation, outer hair cell loss broadens the frequency tuning of cochlear neurons and changes their response phase, which can desynchronize the ensemble neural signal^22,23^. These changes in the way sounds are coded in the cochlear nerve cannot be corrected by amplification, and any loss of cochlear neurons will further degrade the information being transmitted to the central nervous system.

These basic concepts suggest that the discrepancies between predicted and measured word scores should be smaller in those with CHL than those with SNHL of similar magnitude. Furthermore, among those with SNHL of similar magnitude, and thus a similar degree of sensory cell damage, word-score discrepancies should be greatest in those with the most CND. To test these ideas, we analyzed data from nearly 96,000 ears from patients seen at the Massachusetts Eye and Ear, with normal hearing, CHL or SNHL of several etiologies, including age-related hearing loss (ARHL), noise damage, ototoxic drugs, sudden SNHL, Ménière’s disease and vestibular schwannomas. We observed that the WRS discrepancies were largest in those SNHL etiologies associated with the greatest degree of CND, consistent with the idea that loss of cochlear neurons is a major contributor to the loss of speech intelligibility in SNHL.

## Materials and methods

### Study populations

We collected audiological data from a large sample of adult patients who underwent a comprehensive hearing evaluation at the Massachusetts Eye & Ear between 1993 and 2017 (see Table 1). We considered several groups of patients including those with (1) normal hearing thresholds (≤ 20 dB HL across test frequencies, n = 36,855 ears) and (2) ARHL only; i.e., with an idiopathic bilateral and symmetrical SNHL (n = 44,967 ears). All these patients had differences ≤ 10 dB between air-conduction (AC) and bone-conduction (BC) thresholds (air–bone gap) at any test frequency and/or did not have an interaural asymmetry ≥ 10 dB in AC thresholds at 2 consecutive frequencies or 15 dB at one test frequency. As a kind of control for cognitive decline, we added a third group of patients with conductive hearing loss only (n = 7396 ears); i.e., patients with an air–bone gap ≥ 15 dB at any test frequency and normal bone conduction thresholds. To assess the effect of known etiologies with sensorineural deficits we added groups of patients with (1) neuropathy including a diagnosis of vestibular schwannoma (n = 664 ears) or neurofibromatosis of type 2 (n = 282 ears); (2) Ménière’s disease (n = 628 ears without and n = 128 ears with trans-tympanic injection of gentamicin); (4) a history of sudden (S)SNHL (n = 156 ears); (3) exposure to ototoxic drugs including carboplatin or cisplatin (n = 1135 ears), vancomycin (n = 32 ears) or gentamicin (n = 42 ears); and (5) self-report of recreational or occupational noise damage (n = 2091 ears) or presenting with an audiometric 4-kHz notch (n = 1448 ears) with a difference ≥ 20 dB on either test frequency adjacent to 4 kHz. To prevent multiple inclusion of patients, only the first or last visit was considered (Table 1). Only native speakers of English were included.

**Table 1:** Demographics of study populations.

Groups	Subgroups	Visit	n (ears)	Age (years)		Sex (%)	
				Range	Mean $\pm$ SD	Male	Female
Controls	Normal hearing	First	36,855	18–95	40 $\pm$ 12	36	64
	ARHL		44,967	18–103	62 $\pm$ 14	45	55
	Conductive hearing loss		7396	18–88	40 $\pm$ 14	45	55
Neuropathy	Vestibular schwannoma		664	18–87	53 $\pm$ 13	45	55
	Neurofibromatosis type 2		282	18 -76	40 $\pm$ 16	45	55
Noise Exposure	4-kHz notch		1448	18–96	53 $\pm$ 12	77	23
	Self-report		2091	19–91	59 $\pm$ 13	91	9
Idiopathic	Sudden SNHL	Last	156	22–89	60 $\pm$ 14	56	44
Ménière’s Disease	No gentamicin		628	18–95	60 $\pm$ 13	51	49
	Gentamicin (TTI)		128	30–88	59 $\pm$ 13	45	55
Ototoxic	Chemotherapeutics		1135	18–91	58 $\pm$ 17	47	53
	Vancomycin		32	25–90	60 $\pm$ 19	69	31
	Gentamicin (IV)		42	23–86	60 $\pm$ 18	62	38

This study was reviewed and approved by the Institutional Review Board (IRB) of Mass Eye & Ear and all methods were performed in accordance with the relevant guidelines and regulations of our institution. A waiver of informed consent was obtained by the same IRB.

### Hearing evaluation

**Figure 1:**
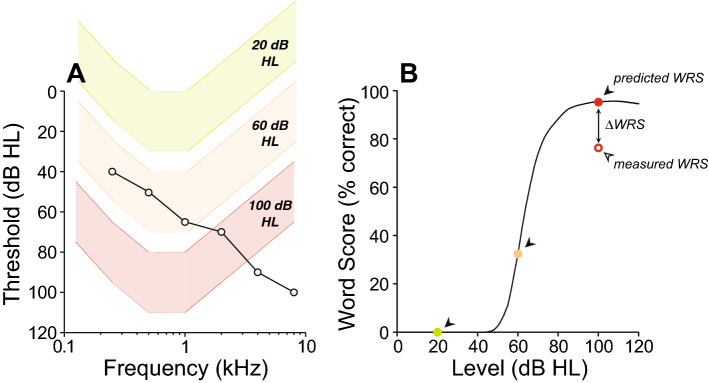
Illustration of the relationship between audiometric thresholds and the performance-intensity function for speech recognition (i.e., speech intelligibility curve). The speech intelligibility curve in (**B**) is drawn from the amount of speech material that is audible to a patient with a specific hearing loss (**A**) combined with a transfer function appropriate to the speech material. In (**A**), W-22 phonetically balanced words presented at either 20 dB HL (green), 60 dB HL (orange) or 100 dB HL (red). To index the loss of intelligibility in word recognition that cannot be accounted by a loss of audibility, we also calculated the difference between the measured word recognition score and the one predicted by the SIC in each patient (**B**).

Audiometric thresholds were obtained using a number of different audiometers including Grason-Stadler (GS-10, GS-16), Interacoustics AC-30, Virtual 320 and Interacoustics Equinox, running under the same Harvard Audiometer Operating System (AOS)^24^. Only pure-tone air-conduction (AC) thresholds measured at standard audiometric frequencies from 0.25 kHz to 8 kHz (in octave steps) using TDH39 headphones or ER-3A insert earphones were considered. Bone-conduction (BC) thresholds were acquired from 250 Hz to 4000 Hz with a Radioear B-71 vibrator over the mastoid. Word recognition was assessed using a recorded CID (Central Institute for the Deaf) W-22 test, consisting of 50 monosyllabic word lists presented with a contralateral masker (speech-shaped noise). The Articulation Index (AI) was used to predict the speech intelligibility curve (SIC, Fig. 1B), a speech intelligibility performance as a function of presentation level based on the audiogram^25,26^ (Fig. 1A), using a transfer function for CID W-22^27^. This procedure was automatically generated by the Harvard AOS software as previously described^28^. The level at which maximal intelligibility was predicted (PB_max_) was chosen as the presentation level. If this value, however, fell below 70 dB HL, presentation level remained at 70 dB HL. To assess word recognition deficits that cannot be accounted by a loss of audibility, we measured either (1) the WRS obtained at predicted PB_max_ and (2) the difference between the measured vs. predicted WRS (∆WRS) as defined by the SIC in each patient (Fig. 1B).

### Statistics

Using Matlab R2018a (The Mathworks, Natick, MA), Pearson correlation coefficients were used to assess relationships between pure-tone thresholds and age. Ordinary least square regression was used to estimate the effect of age on pure-tone thresholds and word recognition scores. Visual inspection, medians and non-parametric Wilcoxon rank-sum tests were used to compare the WRS of groups according to pure-tone thresholds.

To assess the relative contribution of each test frequency to WRS, we designed the following model: WRS or ∆WRS ~ AC_250Hz_ + AC_500Hz_ + AC_1kHz_ + AC_2kHz_ + AC_4kHz_ + AC_8kHz_, whereby AC refers to Air-Conduction threshold. We fitted the regression using the linear model “lm” function in R software environment with the default parameters and the above model. We then computed the respective commonality coefficients and determined the unique and common effects for each predictor variable using R^29^ with the statistical package described by Nimon and col^30^.

To assess the impact of hearing sensitivity at each test frequency on word recognition, we used SAS (SAS Institute, version 9.4) to conduct a principle component analysis (PCA) of the thresholds at each test frequency. These analyses provided a set of 6 linearly independent components as well as the proportion of the total variance in the data that is explained by each component. We use these components as predictors in linear regression models with WRS as outcomes.

Using Excel (Microsoft, version 16.16.27), linear regression analyses were used to investigate the relationship between etiologies and age or hearing loss on outcomes (WRS or ∆WRS). The parameter estimates and associated p-values are detailed in Supplementary Table 1.

The threshold for statistical significance was p = 0.05.

## Results

### The natural history of ARHL

**Figure 2:**
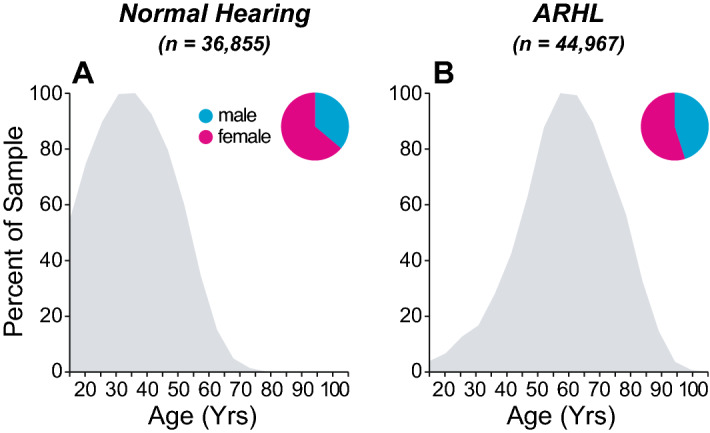
Age distribution of study population split between a group of normal hearing patients (**A**) and one with sensorineural hearing loss (**B**). Pie charts indicate the proportion of male (cyan) vs. female (magenta) for each group.

**Figure 3:**
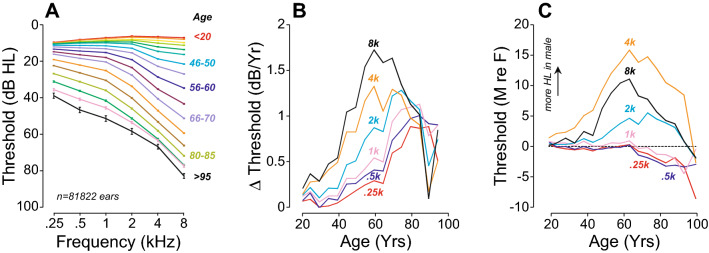
Age effects on hearing thresholds are nonlinear and sex dependent. (**A**) Hearing thresholds at standard audiometric frequencies in study population. (**B**) Progression of hearing loss as a function of age and test frequency. (**C**) Effect of sex on audiometric thresholds as a function of age. Error bars are standard error of mean.

In total, 81,822 ears (59% female, 41% male) from patients aged 18 to 103 years old, presenting with either normal (n = 36,855) or elevated thresholds (n = 44,967) met our inclusion criteria for the normal hearing or ARHL groups, respectively (see Fig. 2, Table 1). Patients with normal hearing were more often female (64%) than those with ARHL (55%). As shown in Fig. 3A, thresholds worsened progressively with age (r = 0.56; p < 0.001 for PTAs), remaining within normal limits until around age 50 and progressing to a down-sloping, moderate to severe loss in our oldest patients. The correlation between age and threshold was stronger at high frequencies (r = 0.74; p < 0.001 for 8 kHz) than low frequencies (r = 0.41; p < 0.001 for 0.25 kHz).

Across all ages, the rate of threshold shift ranged from 0.25 dB/year at 0.25-kHz to 1.12 dB/year at 8-kHz; however, the rate of threshold deterioration also changed with age. As seen in Fig. 3B, threshold shifts increased most rapidly at the highest frequency (8 kHz) until about age 75, after which time any additional threshold shifts were relatively flat across frequency. Correspondingly, threshold shifts at low frequencies (≤ 1 kHz) rose slowly until about age 65, then began to rise rapidly, and more or less in unison, for the next 20 years.

Sex differences in the progression of ARHL are also interesting. Plotting the difference between males and female thresholds as a function of age (Fig. 3C) shows that aging males tend to lose their hearing sensitivity first, in a pattern that peaks at 4 kHz, strongly suggesting an etiology of acoustic overexposure^31–33^. The rate of progression of this 4 kHz notch peaks at age 60, at which time females begin to lose sensitivity faster than males, but only at the three lowest test frequencies.

### Impact of hearing loss configuration on intelligibility deficits

We examined the differences between predicted and measured scores on the standard clinical test of word recognition in quiet. When the measured WRS is lower than the predicted WRS, it means the deficit is not simply one of audibility, and one contributing factor can be loss of auditory nerve fibers, i.e. CND. WRS predictions are derived from the Speech Intelligibility Curve (SIC), which treats a threshold shift like an acoustic filter applied to the words, and quantifies the fraction of the speech spectrum remaining in the filtered output as a function of presentation level^20,25,34^. Because word presentation level (as dictated by the SIC) is well above threshold, a very large degree of hearing loss is needed to reduce the predicted WRS. Accordingly, > 99% of our cases have a predicted word score > 95% correct. In practice at the Massachusetts Eye and Ear, word tests are presented at a level at which the SIC has asymptoted (PB_max_), or at 70 dB HL, whichever is lower.

**Figure 4:**
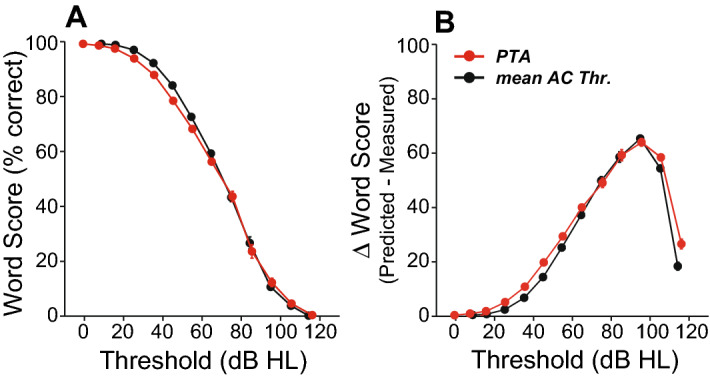
Intelligibility worsens with degree of sensorineural hearing loss. Intelligibility deficits assessed by measuring word recognition scores at PB_max_ (**A**) or by calculating the difference between measured word score and score predicted by the speech intelligibility curve (**B**) show a progression of intelligibility deficits with degree of hearing loss as measured using the pure-tone average (PTA) or mean Air-Conduction thresholds across test frequencies (mean AC Thr.). Error bars are standard error of mean.

The mean WRSs in our normal hearing and ARHL groups declined monotonically (Fig. 4A), in a roughly sigmoidal fashion, with increasing threshold shift, whether expressed as PTA (averaging 0.5–2 kHz) or as mean AC thresholds (averaged across all test frequencies). The WRS discrepancy (predicted vs. measured) rolls over as threshold shift increases (Fig. 4B), because past a certain level of hearing loss, the predicted WRS falls rapidly.

**Figure 5:**
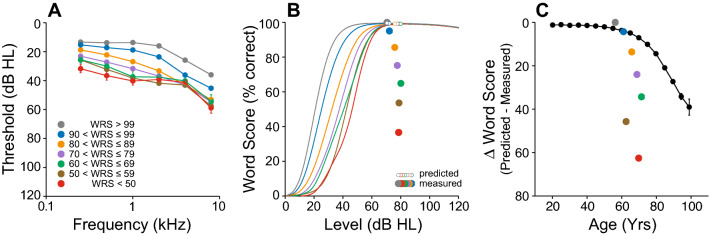
Massive intelligibility deficits can be associated with moderate hearing loss. (**A**) Mean air-conduction thresholds at standard audiometric frequencies from all patients with sensorineural hearing loss whose word recognition performance is predicted ≥ 99% by the speech intelligibility curve as a function of measured word recognition score. (**B**) Speech intelligibility curves for each subgroup of patients described in (**A**) with their associated predicted and measured word recognition score. (**C**) Intelligibility deficits assessed by calculating the difference between measured word score and score predicted by the speech intelligibility curve in each subgroup of patients described in **A** as a function of age. Error bars are standard error of mean.

For one set of analyses, we considered only patients from the normal hearing and ARHL groups with predicted WRSs ≥ 99%, then averaged data according to the measured WRS (Fig. 5). The mean audiogram for those with near-perfect WRSs (grey symbols in Fig. 5A) shows that sensitivity at the highest frequencies is not critical for intelligibility of words in quiet. As the measured WRS declines, thresholds deteriorate, most markedly and steadily at low frequencies, expected to be most important for speech (Fig. 5A). This variation in WRS discrepancy was not related to presentation level (Fig. 5B) or age (Fig. 5C), suggesting that age-related cognitive decline was not a confound in these results. No effect of sex on either relation was found (data not shown).

**Figure 6:**
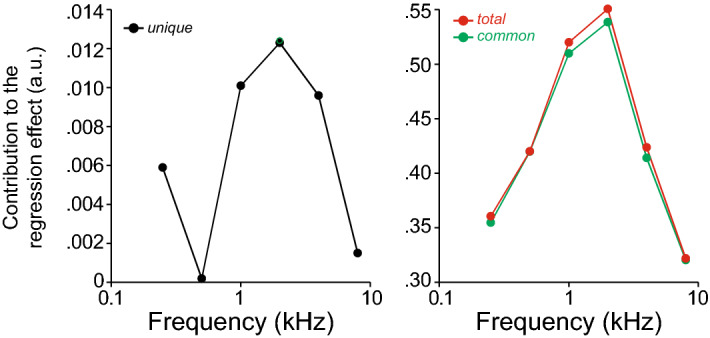
Contribution of cochlear regions to WRS/∆WRS. Commonality coefficients were computed to determine the unique and common effects across all predictors to the explained variance (R-squared) in the regression model.

To further clarify the relative contribution of different cochlear regions to the WRS and ∆WRS, we took two approaches. First, we used a linear regression model to compute commonality coefficients to determine the unique and common contribution of each test frequency to the explained variance (R-squared) in the regression model; i.e., to WRS and ∆WRS (see “Materials and methods”). As shown in Fig. 6, a loss in sensitivity at 1–2 kHz produced the largest impact on WRS and ∆WRS. Second, we conducted a principle component analysis, as summarized in Table 2. The first component, which weights all the frequencies equally (see Table 2, column PC1 of Eigenvectors of the covariance matrix) and thus corresponds to the degree of hearing loss, explains ~ 78% of the variance (see Table 2, Eigenvalues of the covariance matrix). Explained variance rises to ~ 92% if we add the second component, which tracks the slope of the audiogram. Finally, ~ 95% of the variance is explained if we add the third component which tracks the weight of the center vs. adjacent test frequencies (Table 2).

**Table 2:** Principle component analysis on the 6 threshold values of each audiogram followed by the REG procedure with WRS as outcome variable and the 6 principal components as predictors, selected by stepwise regression.

Covariance matrix						
	250 Hz	500 Hz	1000 Hz	2000 Hz	4000 Hz	8000 Hz
250 Hz	1	0.87	0.77	0.66	0.56	0.54
500 Hz	0.87	1	0.89	0.77	0.64	0.61
1000 Hz	0.77	0.89	1	0.87	0.71	0.67
2000 Hz	0.66	0.77	0.87	1	0.85	0.77
4000 Hz	0.56	0.64	0.71	0.85	1	0.88
8000 Hz	0.54	0.61	0.67	0.77	0.88	1

Eigenvalues of the covariance matrix						
	Eigenvalue	Difference	Proportion	Cumulative		
PC 1	4.694	3.951	0.782	0.782		
PC 2	0.743	0.483	0.124	0.916		
PC 3	0.260	0.123	0.043	0.950		
PC 4	0.137	0.042	0.023	0.973		
PC 5	0.095	0.025	0.016	0.988		
PC 6	0.070		0.012	1		

Eigenvectors of the covariance matrix						
	PC 1	PC 2	PC 3	PC 4	PC 5	PC 6
250 Hz	0.382	− 0.524	0.554	0.381	0.294	0.203
500 Hz	0.416	− 0.417	0.005	− 0.327	− 0.488	− 0.555
1000 Hz	0.428	− 0.198	− 0.500	− 0.335	0.020	0.644
2000 Hz	0.429	0.160	− 0.495	0.388	0.456	− 0.433
4000 Hz	0.403	0.477	0.111	0.440	− 0.599	0.212
8000 Hz	0.389	0.508	0.432	− 0.541	0.329	− 0.058

Parameter estimates						
Variable	Degree of freedom	Parameter estimate	Standard error	t value	Pr > |t|	
Intercept	1	3.977	0.023	171.46	< 0.001	
PC 1	1	3.209	0.011	299.80	< 0.001	
PC 2	1	0.090	0.027	3.34	< 0.001	
PC 3	1	− 2.136	0.045	− 47.00	< 0.001	
PC 4	1	1.493	0.063	23.85	< 0.001	
PC 5	1	1.390	0.075	18.47	< 0.001	
PC 6	1	1.317	0.088	15.00	< 0.001	

### Impact of age and cognition on intelligibility deficits

**Figure 7:**
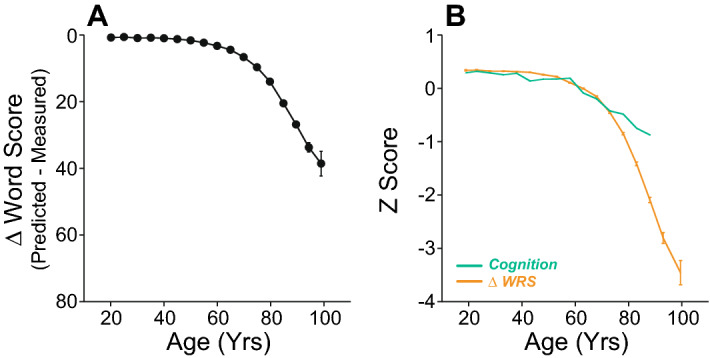
Intelligibility worsens with age independently of cognition. (**A**) Intelligibility deficits assessed as differences between measured and predicted word scores as a function of age. (**B**) Comparison of intelligibility deficits measured in (**A**) with working memory assessed using the Wechsler Adult Intelligence Scale (WAIS) as a function of age. To compare these two tests, data from each have been converted to Z-scores. Error bars are standard error of mean.

Among our subjects with ARHL, intelligibility worsened monotonically with age (Fig. 7A): slowly at first (0.08%/year for those 20–50 years), then more rapidly (0.98%/year for ages over 65). We estimated the contribution of cognitive decline to these trends by comparing our data to published metrics of verbal comprehension, perceptual reasoning, working memory and verbal memory^35^. Converting both to z-score measures as a function of age (Fig. 7B) suggests that the WRS decline is more rapid than general cognitive decline.

**Figure 8:**
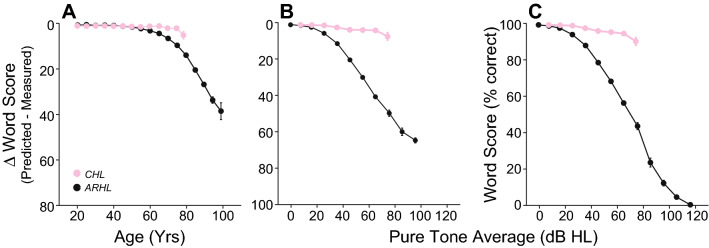
Intelligibility deficits are minimal in patients with conductive hearing loss. (**A**) Intelligibility deficits assessed as differences between measured and predicted words scores as a function of age in patients presenting either a sensorineural hearing loss (SNHL) or a conductive hearing loss (CHL). Legend in (**A**) applies de (**B**) and (**C**). (**B**, **C**) Decline in word recognition score performance as a function of audiometric loss in patients with SNHL (see Fig. 4) are compared to patients presenting with a CHL. Intelligibility deficits were assessed either by calculating the difference between measured word score and score predicted by the speech intelligibility curve (**B**) or by measuring word recognition scores at PB_max_ (**C**). Error bars are standard error of mean.

Further evidence that cognitive decline is not a major confound is seen in the group with CHL, which shows minimal effects of age on WRS (Fig. 8A). Unfortunately, the age range for the CHL group is limited, because most patients past 80 years have at least some SNHL. As confirmation of the utility of the SIC, only a small decline in WRSs was observed in the CHL group with increasing audiometric loss (Fig. 8C), presumably because effects of CHL are indeed similar to those of an acoustic filter. To further support of our interpretation of WRS discrepancies, we compared age-matched patients from the CHL (Supplementary Fig. 1A) and ARHL groups (i.e. 50 years old, the point of overlap of the two age distributions—Supplementary Fig. 1B): as shown in Supplementary Fig. 1C, the WRS discrepancies are minimal and do not rise as the average hearing loss increases.

### Intelligibility deficits in SNHL of other etiologies

**Figure 9:**
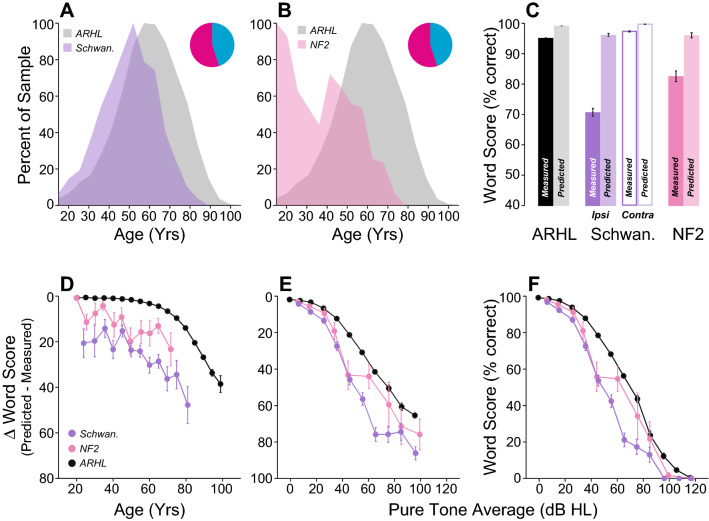
Intelligibility deficits in patients with diagnosed neuropathies are significantly worse than age-matched controls. (**A**, **B**) Age distribution of patients with Vestibular Schwannoma (Schwan.) (**A**) or Neurofibromatosis of type 2 (NF2) (**B**). Pie charts indicate the proportion of male (cyan) vs. female (magenta) for each group. (**C**) Comparison of mean WRS as measured in neuropathic patients or predicted by the speech intelligibility curve. Scores are compared across ears (ipsi vs. contra) and with patients presenting ARHL. (**D**–**F**) Speech Intelligibility deficits as a function of age (**D**) or pure-tone average (**E**, **F**). Legend in (**D**) applies to (**E**–**F**).

If the WRS discrepancy is associated with CND, it should be large in SNHL etiologies where the underlying pathology destroys cochlear nerve fibers. To test this notion, we identified a neuropathy group with either vestibular schwannoma (VS, n = 664 ears) and/or neurofibromatosis of type 2 (NF2, n = 282 ears), both of which are known to cause damage to the cochlear nerve^36^. The VS sample was slightly younger than the ARHL group (Fig. 9A), and the NF2 patients showed a bimodal age distribution at time of test (Fig. 9B), consistent with epidemiologic data^37,38^. As expected, both groups had significantly larger WRS discrepancies compared to the ARHL group (p < 0.001 for both) or to the opposite healthy ear (Fig. 9C, p < 0.001). The differences in ∆WRS for both etiologies were especially striking when plotted as a function of age or hearing loss (Fig. 9D–F, Supplementary Table 1).

**Figure 10:**
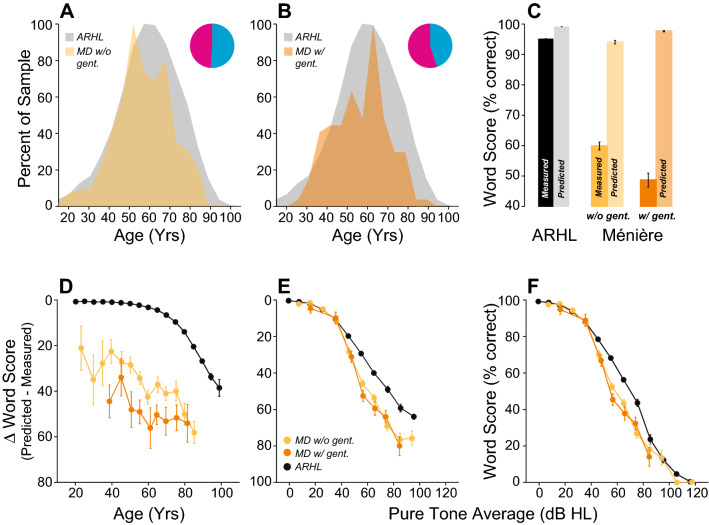
Intelligibility deficits in patients diagnosed with Ménière’s disease are dramatically worse than age-matched controls. (**A**, **B**) Age distribution of patients with Ménière’s disease who received (MD w/ gent., (**B**) or not (MD w/o gent., (**A**) a trans-tympanic injection of gentamicin as part of their treatment. Pie charts indicate the proportion of male (cyan) vs. female (magenta) for each group. (**B**) Comparison of mean WRS as measured in patients with Ménière’s disease or predicted by the speech intelligibility curve. Scores were compared with patients presenting ARHL. (**D**–**F**) Speech Intelligibility deficits as a function of age (**D**) or pure-tone average (**E**, **F**). Legend in (**E**) applies to (**D**, **F**).

We also included a group with Ménière’s disease, because massive CND was reported in a case of unilateral Ménière’s using electron microscopy^39^. In our study, we separated patients who did not receive gentamicin to control vertigo (n = 628 ears) from those who did (n = 128 ears). The Ménière’s groups both had similar age distributions to the ARHL group (Fig. 10A,B), but their WRSs were dramatically poorer (Fig. 10C, p < 0.001 for both groups). When compared as a function of age or hearing loss, both groups had worse word scores than ARHL patients (Fig. 10D–F, Supplementary Table 1). In addition, Ménière’s patients who received gentamicin scored worse than those who did not (Fig. 10C,D, p < 0.001). However, these differences (gentamicin vs. no gentamicin) disappeared when the degree of hearing loss was considered (Fig. 10E,F, p > 0.05), suggesting that differences were related to loss in audibility from gentamicin-induced outer hair cell loss.

**Figure 11:**
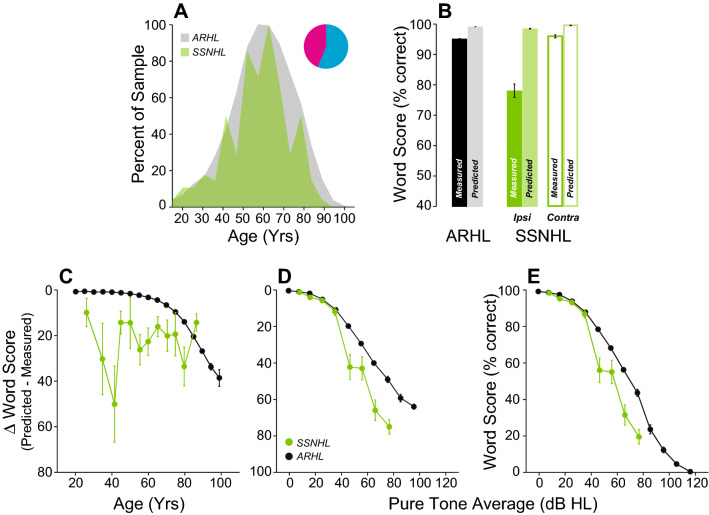
Intelligibility deficits in patients with Sudden Sensorineural Hearing Loss (SSNHL) are significantly worse than age-matched controls. (**A**) Age distribution of patients with SSNHL. Pie chart indicates the proportion of male (cyan) vs. female (magenta). (**B**) Comparison of mean WRS as measured in patients with SSNHL or predicted by the speech intelligibility curve. Scores are compared across ears (ipsi vs. contra) and with patients presenting ARHL. (**C**–**E**) Speech Intelligibility deficits as a function of age (**C**) or pure-tone average (**D**, **E**). Legend in (**D**) applies to (**C**, **E**).

In a recent study, we showed that patients who recovered from idiopathic SSNHL had poorer WRSs than predicted by the residual loss of audibility^40^, and CND is one histopathological feature of SSNHL^41^, as well as of viral infection and ischemia, the main two candidate etiologies of SSNHL^42–46^. Here, we identified a SSNHL group (n = 156 ears) with similar age distribution to the ARHL group (Fig. 11A). SSNHL patients had significantly worse WRS in the affected ear compared to either their own other ear or the ARHL group (Fig. 11B, p < 0.001 for either comparison). Poorer performance remained when considered as a function of age or hearing loss (Fig. 11C–E, Supplementary Table 1).

**Figure 12:**
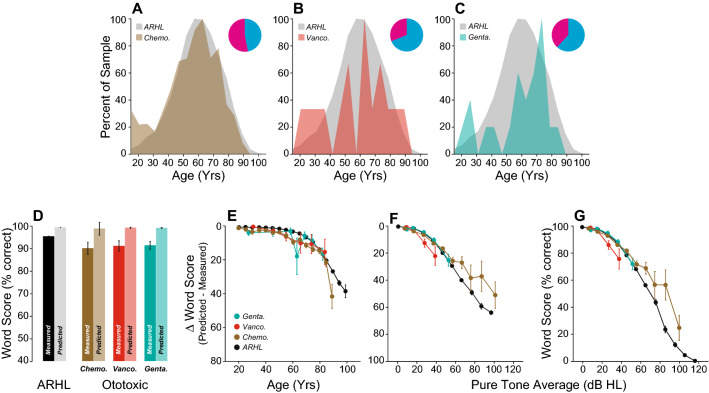
Intelligibility deficits in patients with exposure to ototoxic drugs. (**A**–**C**) Age distribution of patients with exposure to chemotherapeutics (Chemo., **A**), Vancomycin (Vanco., **B**) or I.V. Gentamicin (Genta., **C**). Pie charts indicate the proportion of male (cyan) vs. female (magenta) for each group. (**D**) Comparison of mean WRS as measured in patients exposed to ototoxic drugs or predicted by the speech intelligibility curve. Scores were compared with patients presenting ARHL. (**E**–**G**) Speech Intelligibility deficits as a function of age (**E**) or pure-tone average (**F**, **G**). Legend in (**E**) applies to (**F**, **G**).

To these three SNHL etiologies, we added patients who received systemic ototoxic drugs, either (1) platinum-based cancer therapeutics (cisplatin and carboplatin, n = 1135 ears) or an ototoxic antibiotic, either (2) vancomycin (n = 32 ears), a glycopeptide, or (3) gentamicin (n = 42 ears), an aminoglycoside. All these drugs are known to cause hearing loss, as measured in a standard audiogram^47^. In general, the WRS discrepancies in these groups were similar to those in the normal-aging group. In the gentamicin group, word scores were statistically indistinguishable from patients with age-matched controls (Fig. 12D, p > 0.05), whether considered as a function of age or hearing loss (Fig. 12E–G, Supplementary Table 1). Likewise, ∆WRSs were similar overall in patients treated with vancomycin (Fig. 12D, p > 0.05) including as a function of age (Fig. 12E, Supplementary Table 1); however, significantly poorer word scores were noted when considered as a function of hearing loss (Fig. 12F,G, Supplementary Table 1). In the chemotherapeutics group, both WRS and ∆WRS were poorer overall (Fig. 12D, p < 0.001) and when considered as a function of age (Fig. 12E). Scores were better than the ARHL group when considered as a function of hearing loss (Fig. 12E–G, Supplementary Table 1), perhaps because the drugs destroy outer hair cells, thereby increasing thresholds, without worsening the CND that comes with normal aging.

**Figure 13:**
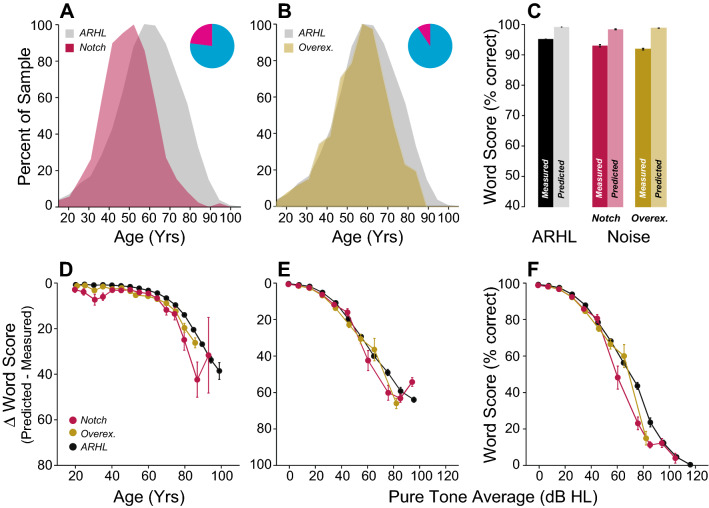
Intelligibility deficits in patients with inferred acoustic overexposure. (**A**, **B**) Age distribution of patients presenting with a 4-kHz audiometric notch (Notch, **A**) or reporting a history of occupational and/or recreational noise overexposure (Overex., **B**). Pie charts indicate the proportion of male (cyan) vs. female (magenta) for each group. (**C**) Comparison of mean WRS as measured in patients with inferred acoustic overexposure or predicted by the speech intelligibility curve. Scores were compared with patients presenting ARHL. (**D**–**F**) Speech Intelligibility deficits as a function of age (**D**) or pure-tone average (**E**, **F**). Legend in (**D**) applies to (**E**, **F**).

Finally, we identified patients with putative noise-induced hearing loss, either presenting with an audiometric notch ≥ 20-dB at 4 kHz (n = 1448 ears) or with an explicit history of occupational or recreational noise exposure (n = 2091 ears). In these younger and male-dominated groups (Fig. 13A,B), ∆WRS was overall significantly poorer only in patients presenting with an audiometric notch (p < 0.001). However, small but significantly poorer WRSs were noted as a function of age or hearing loss when measured with the PTA (Fig. 13D–F, Supplementary Table 1). However, when all test frequencies were considered, deficits in word recognition in patients with self-report of noise exposure was similar to the ARHL group (Supplementary Table 1).

## Discussion

## Effect of age and sex on hearing thresholds

Here, we gathered data on hearing sensitivity from a large number of adult patients with ages covering the lifespan. Our cohort might not reflect hearing status of the general population, because it includes only people who sought the care of an otologist or audiologist. Nevertheless, our value for hearing-loss prevalence (55%) is similar to that in the Beaver Dam study (80%) of a more random sample^48^ if we restrict our age range to match theirs (i.e. 48 to 92 years) and use similar definitions of hearing loss. The overall hearing loss prevalence in the Framingham study was even higher (83%)^49–51^. Clearly, hearing loss is rampant and may be underestimated here, since these studies included more females than males (58% vs. 42% in the present study), and females tend to have better hearing at most ages than males (Fig. 1).

It is worth noting that 45% of patients in our cohort had “normal” hearing. While some of this arises because some of the audiometric referrals were unrelated to an ear or hearing complaint (e.g., a physical exam or a hearing evaluation for a dizziness complaint), this rate emphasizes that many patients report a hearing issue that is not captured by a standard hearing test.

Hearing sensitivity decreased with age and increasing test frequency (Fig. 3), following the classic down-sloping pattern of ARHL (for review, see^52^). At the highest frequencies, threshold decline was significant even from the youngest ages examined (18 yrs), and mean thresholds fell outside the “normal” range (20 dB HL) by age 45, consistent with previous studies^53,54^.

The sloping audiometric pattern of ARHL is consistent with the basal-turn loss of outer hair cell loss in aging humans^3^. However, the most comprehensive histological analyses show prominent apical and basal loss of outer hair cells, with a relative sparing of mid cochlear regions^3,55,56^. Apical OHC degeneration is not well reflected in the audiogram, because (1) the lowest frequency tested (250 Hz) is far (~ 14%) from the apical extreme of the human cochlea^57^, (2) the contribution of OHC electromotility to cochlear amplification decreases with increasing frequency^21^ and (3) low-frequency auditory-nerve fibers are so broadly tuned that apical losses are hidden by neighboring cochlear regions with minimal increase in sound pressure level^58^. Despite the minimal effect on the audiogram, the loss of information due to the silencing of apical neurons must decrease the fidelity of stimulus representation especially in difficult listening environments.

As shown in Fig. 3C, there were significant sex differences in hearing loss progression across the lifespan. Before age 65, loss was more prominent in males, particularly at 4 kHz, a configuration seen in patients with a history of noise exposure^32,59^. Increased hearing loss in males has been linked to occupation, noise exposure history and income level^49,50,60,61^. However, even after adjusting for these risk factors, sex differences remained significant, and smoking, atherosclerosis and other factors have been suggested as possible contributors^48^. Past age 65, hearing loss accelerated at the lower frequencies (≤ 1 kHz), with larger losses in females (Fig. 3C). This low-frequency hearing loss in older women has been associated with vascular disease^62^ and may be related to the degeneration of the stria vascularis, which is worse in apical cochlear region in aging humans, where it would be expected to cause a low-frequency hearing loss^3^.

### Impact of hearing thresholds and cognition on intelligibility

The speech intelligibility index, i.e. the proportion of the total speech information audible to the listener^63^, can be used to predict the WRS, based on the audiogram and the spectrum of the speech tokens used. However, despite the corrections applied to the speech intelligibility curve^64^ to account for upward spread of masking and the negative impact of high presentation levels^65^, the loss of outer hair cells that typically underlies threshold elevation also degrades frequency tuning and critical cochlear non-linearities^22,66^ that can reduce intelligibility. Correspondingly, in a purely conductive hearing loss, where damage to outer hair cells is minimal, the measured and predicted WRSs are quite similar (Fig. 8). Nevertheless, the large range in word scores seen with similar patterns and degrees of mild to moderate SNHL (Fig. 5) argues against an exclusive role for outer hair cell damage in the loss of intelligibility and leaves CND as a prime candidate. The mapping of the hearing loss frequencies on WRS (Fig. 6) and the principle component analysis (Table 2) identify the 1 kHz region as the frequency band most important to the WRS, which is consistent with the peak innervation density in humans^2^, as well as the peak of the spectrum for word tests.

Word recognition is linked to cognition^67^, thus a decline in performance may be related to age-related cognitive decline^35,68^. Our results argue against cognitive factors as a major contributor to the continuous decline in WRS with age given that: (1) deficits in WRS with age are minimal in patients with conductive hearing loss when compared to age-matched patients with sensorineural hearing loss (Fig. 8); (2) age-related cognitive declines are not as steep as the decline in WRS with age (Fig. 7); and (3) WRS is highly variable (< 40% to 100%) in patients of similar age (Fig. 5C).

### Association of intelligibility deficits with CND

We know from animal studies that destruction of cochlear nerve fibers has little impact on audiometric thresholds, until it becomes extreme, i.e. > 80% loss^4–6^. Our working hypothesis is that this CND contributes more prominently to degradation of WRSs, and that differences in the degree of CND are key contributors to the differences in WRSs we observe in different etiologies. Although histopathology of human CND is not extensive, it is informative to consider the present results in light of the available data.

**Figure 14:**
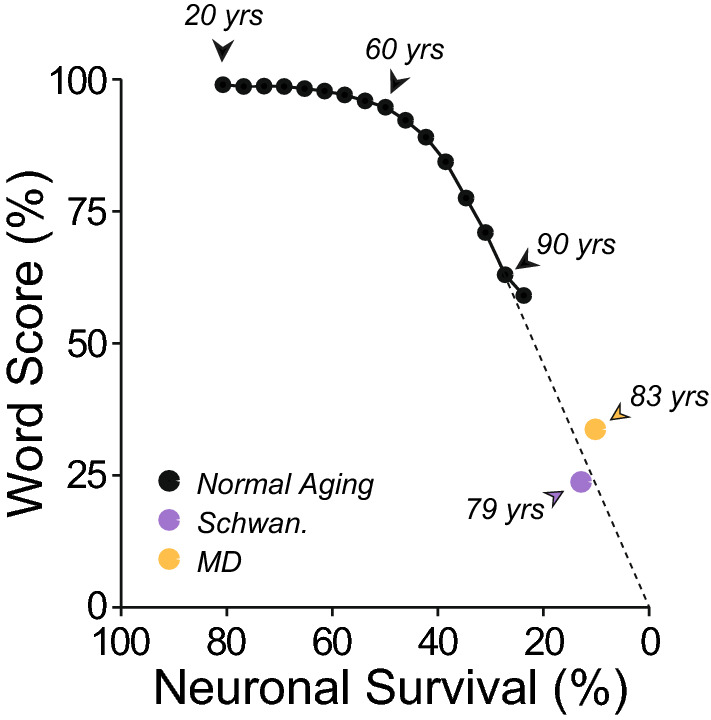
Prediction of word scores as a function of cochlear neural survival. Neuronal survival as a function of age was estimated from a best fit straight line to the data in Fig. 6A from a histopathological study of normal aging humans^3^. Word scores as a function of age were obtained from the present study (Fig. 6A). See text for further details.

Recent work on normal-aging humans shows that cochlear neural degeneration precedes the loss of the inner hair cells they innervate^2,69^. The linear best-fit between age and CND (r^2^ = 0.8) in a sample of 26 cases suggested a mean neural loss of 46% by age 60 years (averaged across all the audiometric frequencies), and an extrapolated loss of 69% by age 90^2^. Referring to the mean WRSs for normal-aging 60 y.o. found here (Fig. 5C), suggests that 46% loss of cochlear nerve fibers has minimal impact (< 5%) on word recognition in quiet, whereas 69% neural loss diminishes word scores by 63%. These two points begin to populate a summary of the estimated relation between word score and CND (black curve in Fig. 14). These inferred relations between CND and WRS suggest an upper bound on the CND contribution, because the WRS discrepancies also include contributions from (1) the degradations in auditory nerve response introduced by loss of frequency tuning, (2) other effects of hair cell damage that are not compensated by raising presentation levels and (3) response alterations in the central auditory pathways elicited by these changes in the periphery^6,12,70,71^. Indeed, the differences in ∆WRSs (Fig. 8A) between the normal-aging cases (with outer hair cell damage) and the CHL cases (without) may provide a measure of the magnitude of that effect.

Patients with explicit neuropathies, i.e. vestibular schwannoma (VS) and/or neurofibromatosis of type 2 (NF2) showed poorer WRSs than the normal-aging group (Fig. 9). VSs are benign tumors in the vestibular nerve that typically arise unilaterally. They can cause hearing impairment, presumably by affecting the blood supply from pressure-induced vascular changes or release of cytokines^36,72,73^. Many VS patients have tinnitus and elevated or absent middle-ear muscle reflexes, both of which have been associated with CND^74–77^. Recent histopathological analysis of temporal bones from five unilateral VS cases (mean age 79 years) revealed a 34% mean loss of the peripheral axons of cochlear nerve fibers in the affected vs. contralateral ears^78^. Assuming the latter are normal for their age, as the present WRS data suggest (Fig. 9C), we estimate 13% survival of cochlear neurons in an 80 y.o. VS patient (i.e. 34% of 39%), which was associated with an average word score of 24%^78^. These estimates fit well on the relation between WRS and CND extrapolated from the normal aging group (Fig. 14).

Of all the etiologies we studied, patients with Ménière’s disease showed the worst WRSs (Fig. 10). This debilitating disorder causes episodic vertigo, fluctuating hearing loss and roaring tinnitus^79,80^, with relatively little loss of either inner or outer hair cells^41^. In an ultrastructural study of a case of unilateral Ménière’s in an 83 year. old, there was a 75% loss of cochlear nerve synapses re the opposite ear, i.e. ~ 3 per inner hair cell on the affected side vs. ~ 12 contralaterally^39^. Given that the normal ear of this 83 year. old had likely lost ~ 65% of cochlear neurons^81^, the affected ear had only about 8% of its original innervation remaining. According to the audiometric record, the WRS in the affected ear of this patient was 34%, compared to a predicted score of 99.3%, based on the speech intelligibility curve and the audiogram. These values also fit well on the summary curve in Fig. 14.

Patients with SSNHL also showed discrepancies between measured and predicted WRSs that were larger than in normal-aging controls (Fig. 11C), but the differences were not as dramatic as those in Ménière’s or VS patients. The etiology of SSNHL is poorly understood, and likely includes a number of underlying causes, including viral infection and cochlear vascular compromise^82,83^. A survey of cochlear histopathology in SSNHL cases with unrecovered thresholds concluded that roughly half (7/15) showed marked CND^41^; however, the neural loss has not been well quantified enough to make any meaningful comparison to the other etiologies.

Of all the etiologies studied, the smallest differences between measured and predicted word scores were seen in cases of ototoxic drugs (Fig. 12) and putative noise-induced damage (Fig. 13). We found no relevant quantitative data in the literature on CND after hearing loss from ototoxic drugs; however, a recent comparison of human temporal bones with vs. without a history of noise damage^3^ found a small increase in CND (~ 15%) across all audiometric frequencies in the latter, but only among patients aged 50–74 years and not among individuals from 75 to 100 years. This age effect is opposite to that seen here, where differences from normal-aging were maximal in the older group. However, both studies agree that the added effects of noise on CND in the aging ear are small. Furthermore, both studies acknowledge the difficulties in identifying normal-aging humans who have not suffered ear abuse, and in relying on self-report as a reliable metric of cumulative noise exposure. That problem is evidenced here by the presence of a 4 kHz notch in the mean audiograms of the normal-aging group (Fig. 3), for which a history of acoustic overexposure was not noted. Nevertheless, the histopathological study showed that including the degree of CND in a multivariable linear regression of histopathological metrics, including hair cell loss and strial damage, helped predict the measured WRS.

## Summary and conclusion

We examined audiometric thresholds, word recognition in quiet and the differences between predicted and measured word scores in nearly 96,000 ears with either normal hearing, ARHL (i.e., idiopathic) or SNHL attributable to diagnosable disease or cochlear insult of several etiologies. Intelligibility worsened monotonically with age and degree of hearing loss. Results from patients with conductive hearing loss suggested that cognitive decline was not a major confound. In SNHL, larger intelligibility deficits were observed in etiologies known, or suspected, to cause greater cochlear nerve loss. After accounting for age and degree of hearing loss, word performance deficits were greatest (in descending order) in Ménière’s disease, in those with acoustic tumors and after sudden sensorineural hearing loss. Intelligibility deficits were considerably smaller in those with a history of exposure to noise or ototoxic drugs (either aminoglycosides or platinum-containing chemotherapeutics). This ranking is consistent with the fragmentary data on CND in human temporal bones. A compilation of those data suggests that auditory nerve loss must exceed 60% before word scores in quiet fall below 90%. The relation between CND and performance on more difficult listening tasks could be much steeper.

These findings agree with a number of studies linking speech perception or signal-in-noise detection with neural deficits measures by ABRs/electrocochleography^84–87^, middle-ear muscle reflex^76,77^, envelope following responses^88^, in vivo imaging of auditory nerve diameter^89^ and computational models^13^. While the evidence implicates CND in the intelligibility challenges of hearing impairment, CND also likely serves as a peripheral trigger for excess central gain and other maladaptive plasticity in central auditory pathways that further degrade perceptual accuracy for complex sounds beyond that predicted by viewing the audiogram as a simple acoustic filter^12,70,90^.

## Supplementary Information

Supplementary Figure 1.

Supplementary Table 1.

## Data Availability

All relevant data are within the paper and its Supporting Information files. Raw data that support the findings of this study are available from the corresponding author, upon reasonable request.

## References

[1] Kujawa SG, Liberman MC (2009). Adding insult to injury: Cochlear nerve degeneration after "temporary" noise-induced hearing loss. J. Neurosci..

[2] Wu PZ (2019). Primary neural degeneration in the human cochlea: Evidence for hidden hearing loss in the aging ear. Neuroscience.

[3] Wu PZ, O’Malley JT, de Gruttola V, Liberman MC (2021). Primary neural degeneration in noise-exposed human cochleas: Correlations with outer hair cell loss and word-discrimination scores. J. Neurosci..

[4] Woellner RC, Schuknecht HF (1955). Hearing loss from lesions of the cochlear nerve: An experimental and clinical study. Trans. Am. Acad. Ophthalmol. Otolaryngol..

[5] Lobarinas E, Salvi R, Ding D (2013). Insensitivity of the audiogram to carboplatin induced inner hair cell loss in chinchillas. Hear. Res..

[6] Chambers AR (2016). Central gain restores auditory processing following near-complete cochlear denervation. Neuron.

[7] Schmiedt RA, Mills JH, Boettcher FA (1996). Age-related loss of activity of auditory-nerve fibers. J. Neurophysiol..

[8] Furman AC, Kujawa SG, Liberman MC (2013). Noise-induced cochlear neuropathy is selective for fibers with low spontaneous rates. J. Neurophysiol..

[9] Costalupes JA, Young ED, Gibson DJ (1984). Effects of continuous noise backgrounds on rate response of auditory nerve fibers in cat. J. Neurophysiol..

[10] Felder E, Schrott-Fischer A (1995). Quantitative evaluation of myelinated nerve fibres and hair cells in cochleae of humans with age-related high-tone hearing loss. Hear. Res..

[11] Monaghan JJM, Garcia-Lazaro JA, McAlpine D, Schaette R (2020). Hidden hearing loss impacts the neural representation of speech in background noise. Curr. Biol..

[12] Resnik J, Polley DB (2021). Cochlear neural degeneration disrupts hearing in background noise by increasing auditory cortex internal noise. Neuron.

[13] Buran BN, McMillan GP, Keshishzadeh S, Verhulst S, Bramhall NF (2022). Predicting synapse counts in living humans by combining computational models with auditory physiology. J. Acoust. Soc. Am..

[14] Bramhall NF, McMillan GP, Gallun FJ, Konrad-Martin D (2019). Auditory brainstem response demonstrates that reduced peripheral auditory input is associated with self-report of tinnitus. J. Acoust. Soc. Am..

[15] Wojtczak M, Beim JA, Oxenham AJ (2017). Weak middle-ear-muscle reflex in humans with noise-induced tinnitus and normal hearing may reflect cochlear synaptopathy. ENeuro.

[16] Hickox AE, Liberman MC (2014). Is noise-induced cochlear neuropathy key to the generation of hyperacusis or tinnitus?. J. Neurophysiol..

[17] Schaette R, McAlpine D (2011). Tinnitus with a normal audiogram: Physiological evidence for hidden hearing loss and computational model. J. Neurosci..

[18] Lewis RM, Jahn KN, Parthasarathy A, Goedicke WB, Polley DB (2020). Audiometric predictors of bothersome tinnitus in a large clinical cohort of adults with sensorineural hearing loss. Otol. Neurotol..

[19] Thornton AR, Raffin MJ (1978). Speech-discrimination scores modeled as a binomial variable. J. Speech Hear. Res..

[20] Boothroyd A (2008). The performance/intensity function: An underused resource. Ear Hear..

[21] Liberman MC (2002). Prestin is required for electromotility of the outer hair cell and for the cochlear amplifier. Nature.

[22] Liberman MC, Dodds LW (1984). Single-neuron labeling and chronic cochlear pathology. III. Stereocilia damage and alterations of threshold tuning curves. Hear. Res..

[23] Liberman MC, Kiang NY (1984). Single-neuron labeling and chronic cochlear pathology. IV. Stereocilia damage and alterations in rate- and phase-level functions. Hear. Res..

[24] The Harvard Audiometer Operating System (Applitech, Inc., 1994).

[25] Pavlovic CV, Studebaker GA, Sherbecoe RL (1986). An articulation index based procedure for predicting the speech recognition performance of hearing-impaired individuals. J. Acoust. Soc. Am..

[26] Wilde G, Humes LE (1990). Application of the articulation index to the speech recognition of normal and impaired listeners wearing hearing protection. J. Acoust. Soc. Am..

[27] Sherbecoe RL, Studebaker GA (1990). Regression equations for the transfer functions of ANSI S3.5–1969. J. Acoust. Soc. Am..

[28] Halpin C, Thornton A, Hasso M (1994). Low-frequency sensorineural loss: clinical evaluation and implications for hearing aid fitting. Ear. Hear..

[29] Team, R. C. *A Language and Environment for Statistical Computing*. https://www.R-project.org/ (2019).

[30] Nimon K, Lewis M, Kane R, Haynes RM (2008). An R package to compute commonality coefficients in the multiple regression case: An introduction to the package and a practical example. Behav. Res. Methods.

[31] Rabinowitz PM (2006). Audiogram notches in noise-exposed workers. Ear. Hear..

[32] Lie A, Engdahl B, Hoffman HJ, Li CM, Tambs K (2017). Occupational noise exposure, hearing loss, and notched audiograms in the HUNT Nord-Trondelag hearing loss study, 1996–1998. Laryngoscope.

[33] McBride DI, Williams S (2001). Audiometric notch as a sign of noise induced hearing loss. Occup. Environ. Med..

[34] Studebaker GA, Gilmore C, Sherbecoe RL (1993). Performance-intensity functions at absolute and masked thresholds. J. Acoust. Soc. Am..

[35] Salthouse TA (2009). Decomposing age correlations on neuropsychological and cognitive variables. J. Int. Neuropsychol. Soc..

[36] Ren Y, Chari DA, Vasilijic S, Welling DB, Stankovic KM (2021). New developments in neurofibromatosis type 2 and vestibular schwannoma. Neurooncol. Adv..

[37] Evans DG (2009). Neurofibromatosis type 2 (NF2): A clinical and molecular review. Orphanet. J. Rare Dis..

[38] Cioffi G (2020). Epidemiology of vestibular schwannoma in the United States, 2004–2016. Neurooncol. Adv..

[39] Nadol JB, Thornton AR (1987). Ultrastructural findings in a case of Meniere's disease. Ann. Otol. Rhinol. Laryngol..

[40] Okada M, Parthasarathy A, Welling DB, Liberman MC, Maison SF (2021). Idiopathic sudden sensorineural hearing loss: Speech intelligibility deficits following threshold recovery. Ear Hear..

[41] Merchant SN, Nadol JB (2010). Schuknecht’s Pathology of the Ear.

[42] Lindsay JR (1973). Histopathology of deafness due to postnatal viral disease. Arch. Otolaryngol..

[43] Linthicum FH (1978). Viral causes of sensorineural hearing loss. Otolaryngol. Clin. N. Am..

[44] Sawada M (1979). Electrocochleography of ears with mumps deafness. Arch. Otolaryngol..

[45] Puel JL, Pujol R, Tribillac F, Ladrech S, Eybalin M (1994). Excitatory amino acid antagonists protect cochlear auditory neurons from excitotoxicity. J. Comp. Neurol..

[46] Suryadevara AC, Schulte BA, Schmiedt RA, Slepecky NB (2001). Auditory nerve fibers in young and quiet-aged gerbils: Morphometric correlations with endocochlear potential. Hear. Res..

[47] Frisina RD (2016). Comprehensive audiometric analysis of hearing impairment and tinnitus after cisplatin-based chemotherapy in survivors of adult-onset cancer. J. Clin. Oncol..

[48] Cruickshanks KJ (1998). Prevalence of hearing loss in older adults in Beaver Dam, Wisconsin: The epidemiology of hearing loss study. Am. J. Epidemiol..

[49] Gates GA, Cooper JC, Kannel WB, Miller NJ (1990). Hearing in the elderly: The Framingham cohort, 1983–1985. Part I. Basic audiometric test results. Ear Hear..

[50] Moscicki EK, Elkins EF, Baum HM, McNamara PM (1985). Hearing loss in the elderly: An epidemiologic study of the Framingham Heart Study Cohort. Ear Hear..

[51] Parthasarathy A, Romero Pinto S, Lewis RM, Goedicke W, Polley DB (2020). Data-driven segmentation of audiometric phenotypes across a large clinical cohort. Sci. Rep..

[52] Eckert MA (2021). Translational and interdisciplinary insights into presbyacusis: A multidimensional disease. Hear. Res..

[53] Gates GA, Mills JH (2005). Presbycusis. Lancet.

[54] Gordon-Salant S (2005). Hearing loss and aging: New research findings and clinical implications. J. Rehabil. Res. Dev..

[55] Bredberg G (1968). Cellular pattern and nerve supply of the human organ of Corti. Acta Otolaryngol..

[56] Keithley EM (2020). Pathology and mechanisms of cochlear aging. J. Neurosci. Res..

[57] Greenwood DD (1990). A cochlear frequency-position function for several species–29 years later. J. Acoust. Soc. Am..

[58] Kiang NY, Moxon EC (1974). Tails of tuning curves of auditory-nerve fibers. J. Acoust. Soc. Am..

[59] Lie A (2016). Occupational noise exposure and hearing: A systematic review. Int. Arch. Occup. Environ. Health.

[60] Corso JF (1963). Age and sex differences in pure-tone thresholds: Survey of hearing levels from 18 to 65 years. Arch Otolaryngol..

[61] Siegelaub AB, Friedman GD, Adour K, Seltzer CC (1974). Hearing loss in adults: Relation to age, sex, exposure to loud noise, and cigarette smoking. Arch. Environ. Health.

[62] Eckert MA (2013). White matter hyperintensities predict low frequency hearing in older adults. J. Assoc. Res. Otolaryngol..

[63] Kringlebotn M (1999). A graphical method for calculating the speech intelligibility index and measuring hearing disability from audiograms. Scand. Audiol.

[64] ANSI. Vol. ANSI S3.5-1997 (1997).

[65] Hornsby BW, Ricketts TA (2006). The effects of hearing loss on the contribution of high- and low-frequency speech information to speech understanding. II. Sloping hearing loss. J. Acoust. Soc. Am..

[66] Schmiedt RA (1984). Acoustic injury and the physiology of hearing. J. Acoust. Soc. Am..

[67] Fullgrabe C, Moore BC, Stone MA (2014). Age-group differences in speech identification despite matched audiometrically normal hearing: Contributions from auditory temporal processing and cognition. Front. Aging Neurosci..

[68] Salthouse TA (2019). Trajectories of normal cognitive aging. Psychol. Aging.

[69] Viana LM (2015). Cochlear neuropathy in human presbycusis: Confocal analysis of hidden hearing loss in post-mortem tissue. Hear. Res..

[70] Parthasarathy A, Hancock KE, Bennett K, DeGruttola V, Polley DB (2020). Bottom-up and top-down neural signatures of disordered multi-talker speech perception in adults with normal hearing. Elife.

[71] Pienkowski M (2017). On the etiology of listening difficulties in noise despite clinically normal audiograms. Ear Hear..

[72] Katsumi S (2019). Intracochlear perfusion of tumor necrosis factor-alpha induces sensorineural hearing loss and synaptic degeneration in guinea pigs. Front. Neurol..

[73] Van Dijk JE, Duijndam J, Graamans K (2000). Acoustic neuroma: Deterioration of speech discrimination related to thresholds in pure-tone audiometry. Acta Otolaryngol..

[74] Carlson ML, Link MJ (2021). Vestibular schwannomas. N. Engl. J. Med..

[75] Valero MD, Hancock KE, Liberman MC (2015). The middle ear muscle reflex in the diagnosis of cochlear neuropathy. Hear. Res..

[76] Mepani AM (2020). Middle ear muscle reflex and word recognition in "normal-hearing" adults: evidence for cochlear synaptopathy?. Ear. Hear..

[77] Shehorn J, Strelcyk O, Zahorik P (2020). Associations between speech recognition at high levels, the middle ear muscle reflex and noise exposure in individuals with normal audiograms. Hear. Res..

[78] Eggink MC (2022). Human vestibular schwannoma reduces density of auditory nerve fibers in the osseous spiral lamina. Hear. Res..

[79] Merchant SN, Rauch SD, Nadol JB (1995). Meniere's disease. Eur. Arch. Otorhinolaryngol..

[80] Rauch SD (2016). Meniere's disease: Damaged hearing but reduced vertigo. Lancet.

[81] Nadol JB (1988). Application of electron microscopy to human otopathology: Ultrastructural findings in neural presbycusis, Meniere's disease and Usher's syndrome. Acta Otolaryngol..

[82] Merchant SN, Adams JC, Nadol JB (2005). Pathology and pathophysiology of idiopathic sudden sensorineural hearing loss. Otol. Neurotol..

[83] Okada M (2017). The effect of initial treatment on hearing prognosis in idiopathic sudden sensorineural hearing loss: A nationwide survey in Japan. Acta Otolaryngol..

[84] Bramhall N, Ong B, Ko J, Parker M (2015). Speech perception ability in noise is correlated with auditory brainstem response wave I amplitude. J. Am. Acad. Audiol..

[85] Liberman MC, Epstein MJ, Cleveland SS, Wang H, Maison SF (2016). Toward a differential diagnosis of hidden hearing loss in humans. PLoS ONE.

[86] Ridley CL, Kopun JG, Neely ST, Gorga MP, Rasetshwane DM (2018). Using thresholds in noise to identify hidden hearing loss in humans. Ear Hear..

[87] Grant KJ (2020). Electrophysiological markers of cochlear function correlate with hearing-in-noise performance among audiometrically normal subjects. J. Neurophysiol..

[88] Mepani AM (2021). Envelope following responses predict speech-in-noise performance in normal hearing listeners. J. Neurophysiol..

[89] Harris KC (2021). Neural presbyacusis in humans inferred from age-related differences in auditory nerve function and structure. J. Neurosci..

[90] Oxenham AJ (2016). Predicting the perceptual consequences of hidden hearing loss. Trends Hear..

