# Supplementary material for: Predicting neural deficits in sensorineural hearing loss from word recognition scores
Source: Sci Rep. 2022 Jun 23;12:8929. doi: 10.1038/s41598-022-13023-5 (PMC9226113; doi:10.1038/s41598-022-13023-5)
Supplement: Supplementary file 1 — Supplementary Figure 1. [file 41598_2022_13023_MOESM1_ESM.pdf]

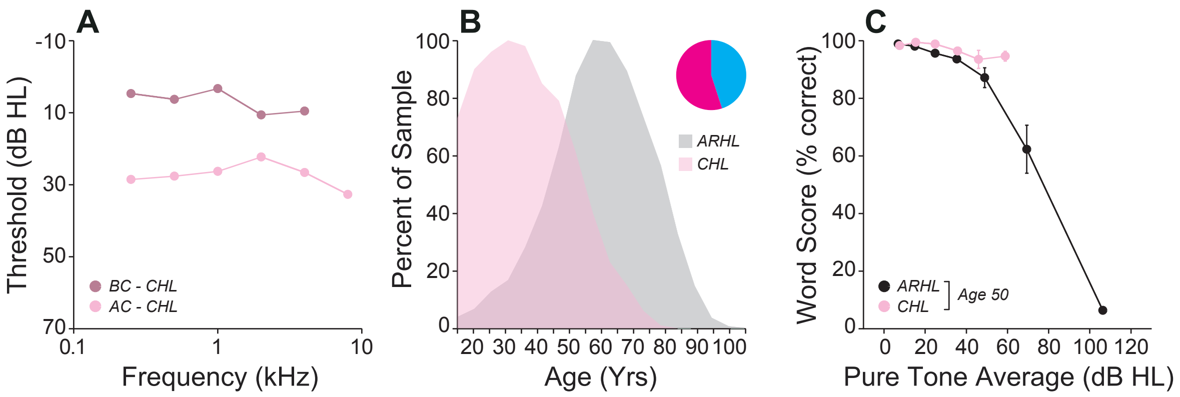

Supplementary Figure 1: A: Mean air-conduction (AC) and bone-conduction (BC) thresholds at standard audiometric frequencies in a group of patients with conductive hearing loss (CHL). B: Comparison of Age distribution in patients with sensorineural hearing loss (SNHL) vs. conductive hearing loss (CHL). Pie chart indicates the proportion of male (cyan) vs. female (magenta). Error bars are standard error of mean. C: Deficits in word recognition performance assessed at PBmax as a function of degree of hearing loss in all 50 y.o. patients from the CHL vs. SNHL groups. Error bars are standard error of mean.
