# Supplementary material for: Predicting neural deficits in sensorineural hearing loss from word recognition scores
Source: Sci Rep. 2022 Jun 23;12:8929. doi: 10.1038/s41598-022-13023-5 (PMC9226113; doi:10.1038/s41598-022-13023-5)
Supplement: Supplementary file 2 — Supplementary Table 1. [file 41598_2022_13023_MOESM2_ESM.docx]

| ARHL vs. | | Age | | PTA | | AC Thr. | |
| --- | --- | --- | --- | --- | --- | --- | --- |
|  |  | WRS | ΔWRS | WRS | ΔWRS | WRS | ΔWRS |
| Neuropathy | VS | -24.0*** | 21.2*** | -14.3*** | 12.9*** | -15.2*** | 13.6*** |
|  | NF2 | -16.3*** | 13.2*** | -7.9*** | 5.3*** | -9.9*** | 7.0*** |
| Ménière’s Disease | w/o gentamicin | -37.5*** | 35.5*** | -16.6*** | 18.1*** | -17.6*** | 18.8*** |
|  | w/ gentamicin | -44.3*** | 42.9*** | -20.1*** | 22.6*** | -20.7*** | 23.1*** |
| Idiopathic | SSNHL | -15.4*** | 15.0*** | -8.0*** | 8.8*** | -8.0*** | 8.7*** |
| Ototoxic | gentamicin | -1.2 ^NS^ | 1.3 ^NS^ | 0.1 ^NS^ | 0.2 ^NS^ | 0.5 ^NS^ | -0.2 ^NS^ |
|  | vancomycin | -1.5 ^NS^ | 1.7 ^NS^ | -3.7* | 3.4* | -3.3* | 3.1* |
|  | carboplatin/cisplatin | -3.2*** | 3.0*** | -1.0*** | 1.0*** | -0.6* | 0.7** |
| Noise Exposure | 4 kHz notch | -1.7*** | 1.2*** | -3.1*** | 2.0*** | -3.2*** | 2.0*** |
|  | self-report | -1.0*** | 0.9*** | -1.6*** | 1.3*** | -0.3 ^NS^ | 0.2 ^NS^ |

**Supplementary Table 1**: Linear regression analyses performed to investigate group differences as a function of age or hearing loss with WRS or ΔWRS as outcome variables. Each cell indicates the parameter estimate and the associated p-value. Level of significance: *p<0.05, **p<0.01, ***p<0.001. NS: not significant.
